# findGSEP: estimating genome size of polyploid species using *k*-mer frequencies

**DOI:** 10.1093/bioinformatics/btae647

**Published:** 2024-10-30

**Authors:** Laiyi Fu, Yanxin Xie, Shunkang Ling, Ying Wang, Binzhong Wang, Hejun Du, Qinke Peng, Hequan Sun

**Affiliations:** School of Automation Science and Engineering, Faculty of Electronic and Information Engineering, Xi’an Jiaotong University, Xi’an 710049, China; Research Institute of Xi’an Jiaotong University, Zhejiang, Hangzhou 311200, China; Sichuan Digital Economy Industry Development Research Institute, Chengdu 610036, China; School of Automation Science and Engineering, Faculty of Electronic and Information Engineering, Xi’an Jiaotong University, Xi’an 710049, China; College of Mechanical and Electrical Engineering, Shihezi University, Shihezi 832000, China; School of Automation Science and Engineering, Faculty of Electronic and Information Engineering, Xi’an Jiaotong University, Xi’an 710049, China; Hubei Key Laboratory of Three Gorges Project for Conservation of Fishes, Yichang, Hubei 443100, China; Hubei Key Laboratory of Three Gorges Project for Conservation of Fishes, Yichang, Hubei 443100, China; School of Automation Science and Engineering, Faculty of Electronic and Information Engineering, Xi’an Jiaotong University, Xi’an 710049, China; School of Automation Science and Engineering, Faculty of Electronic and Information Engineering, Xi’an Jiaotong University, Xi’an 710049, China; Department of Chromosome Biology, Max Planck Institute for Plant Breeding Research, Cologne 50829, Germany

## Abstract

**Summary:**

Estimating genome size using *k*-mer frequencies, which plays a fundamental role in designing genome sequencing and analysis projects, has remained challenging for polyploid species, i.e., ploidy *p* > 2. To address this, we introduce “findGSEP,” which is designed based on iterative curve fitting of *k*-mer frequencies. Precisely, it first disentangles up to *p* normal distributions by analyzing *k*-mer frequencies in whole genome sequencing of the focal species. Second, it computes the sizes of genomic regions related to 1∼*p* (homologous) chromosome(s) using each respective curve fitting, from which it infers the full polyploid and average haploid genome size. “findGSEP” can handle any level of ploidy *p*, and infer more accurate genome size than other well-known tools, as shown by tests using simulated and real genomic sequencing data of various species including octoploids.

**Availability and implementation:**

“findGSEP” was implemented as a web server, which is freely available at http://146.56.237.198:3838/findGSEP/. Also, “findGSEP” was implemented as an *R* package for parallel processing of multiple samples. Source code and tutorial on its installation and usage is available at https://github.com/sperfu/findGSEP.

## 1 Introduction

Many economically important species are polyploids, such as hexaploid wheat (*Triticum aestivum*), tetraploid potato (*Solanum tuberosum spp. tuberosum*), and octoploid strawberry (*Fragaria × ananassa*) (Hirakawa *et al.* 2014, Zimin *et al.* 2017, Sun *et al.* 2022), contributing to human diets worldwide. To understand the genome evolution of such species, a cost-effective sequencing project is required nowadays, where a key step is to estimate the genome size.

Genome size is used to be estimated by wet-lab techniques, such as flow cytometry and Feulgen microdensitometry, which deliver relative genome size as compared to an internal or external reference standard (McKinnon 2018). Therefore, the accuracy in estimates is highly depending on the reference (Dixon and Stead 1977, Doležel *et al.* 1998). Thanks to the advancement in genome sequencing technologies with decreasing costs, *k*-mer frequency analysis has become a popular way for estimating genome size, where a *k*-mer is substring of length *k* extracted from a longer DNA sequence or sequencing reads. Each *k*-mer can be associated with a coverage value, which is its occurrence within the sequencing reads (Pflug *et al.* 2020). By counting how many *k*-mers (i.e. frequency or count *f_i_*) occurring at a coverage of *c_i_*, a *k*-mer frequency distribution histogram, i.e. (*c_i_*, *f_i_*), *i* = 1,2,3,…, can be built up (Al-Qurainy *et al.* 2021). When examining the *k*-mer frequency distribution plot for a homozygous species, the distribution of *k*-mer frequencies tends to resemble a “Poisson” distribution with the peak centered on the average *k*-mer sequencing depth of the genome, while there is another peak next to *y*-axis representing sequence errors (Li and Waterman 2003). For heterozygous species, multiple peaks can occur depending on the heterozygosity and ploidy of the genome (Supplementary Figs S1–S14).

Several *k*-mer analysis tools, such as “CoVest” (Hozza *et al.* 2015), “Kmergenie” (Chikhi and Medvedev 2014), “findGSE” (Sun *et al.* 2018), and “GenomeScope” and “GenomeScope” v2.0 (Vurture *et al.* 2017, Ranallo-Benavidez *et al.* 2020), have been developed for genome size estimation. However, “findGSE” is only capable of estimating genome size for diploid species due to that its curve fitting is generally performed three times (around heterozygous peak, homozygous peak, and repeats), while only “GenomeScope” v2.0, which uses a mixture modeling approach, can deal with polyploid genomes, but the ploidy is restricted up to six.

Here, we introduce “findGSEP,” which is an upgraded version of “findGSE” with two enhanced features. First, it employs a curve-fitting approach to estimate genome size using *k*-mer frequencies, which is the same as “findGSE” but with *p* iterations. Within each iteration, it determines the size of genomes shared by 1, 2, 3, …, *p* homologous chromsomes, which delivers more insights into the polyploid genome regarding haplotype sharing than “findGSE.” In short, “findGSEP” is capable of handling species with any ploidy, from diploid to polyploid species, including octoploids, while “findGSE” only works for diploid species. Second, it is implemented in both a web application and a standalone R package, making it more accessible for different user preferences. The performance of “findGSEP” was evaluated on both simulated and real datasets, as compared with the popular tool “GenomeScope” v2.0 (referred to as “GenomeScope” afterwards). Tests on real data demonstrated that “findGSEP” had better performance across a diverse array of diploid and polyploid species than “GenomeScope,” especially for those with larger genomes or higher ploidy. Simulated data with true genome size showed that “findGSEP” can deliver highly accurate estimations.

## 2 Implementation

**Figure 1.**
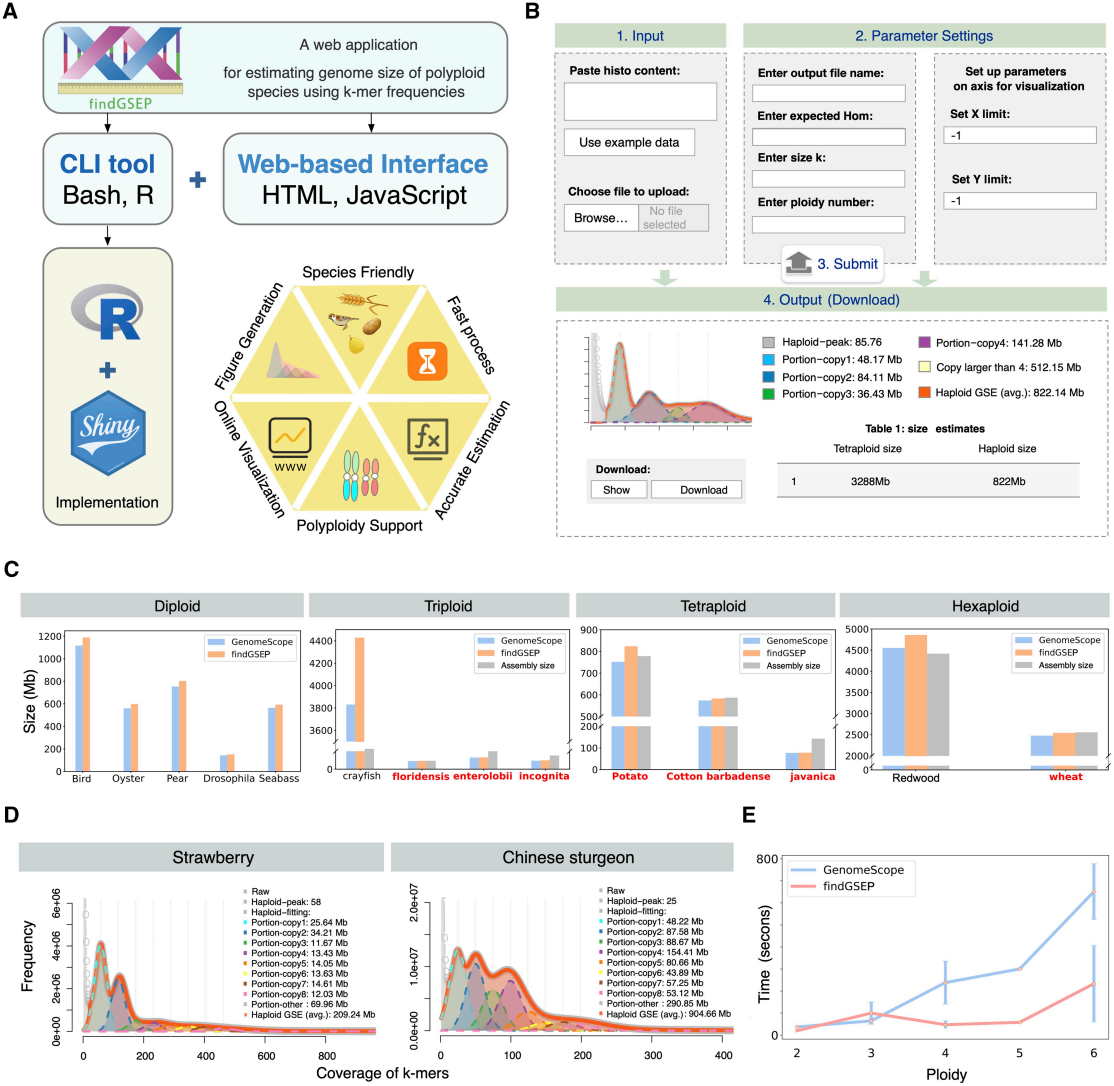
Overview of ‘findGSEP’ and its performance evaluation. (**A**) ‘findGSEP’ offers a command-line *R* package and a web-based platform built up using ‘Shiny’, ‘HTML’, and ‘JavaScript’. (**B**) The online web server requires only four simple steps. First, enter *k*-mer frequency histogram or upload the respective file. Second, specify parameters on the genome of the species as well as visualization, i.e. value of *k*, *k*-mer coverage, ploidy of species, and range of *x*/*y* axis to draw plots. Third, submit the job, where the backend server will subsequently generate estimated genome sizes along with fitted curve plots. Fourth, download the result in a pdf file. (**C**) Performance evaluation of ‘findGSEP’ using species with diverse ploidy levels from diploid to hexaploidy, as compared with estimates given by ‘GenomeScope’ and the respective genome assembly sizes. In most cases (seven out of nine), ‘findGSEP’ generally provides estimation that are relatively closer to the assembly size than ‘GenomeScope’, as colored in red. (**D**) Specifically, for species with ploidy larger than six, e.g. for the octoploid strawberry and Chinese sturgeon, only ‘findGSEP’ can deliver an accurate estimate. (**E**) Time efficiency: ‘findGSEP’ was running faster than ‘GenomeScope’, where the error bar represents measurements taken from multiple species having the same ploidy.

“findGSEP” was implemented as an open-source command-line *R* package and a user-friendly web interface built on the “shiny” framework (https://github.com/sperfu/findGSEP-web). Source code, installation and usage of the *R* package are given at GitHub: https://github.com/sperfu/findGSEP. We have also provided a complete vignette with example data in the *R* package for users to test. The usage of CLI (command-line interface) follows the same pipeline as the web service. Here, for simplicity, we choose the web platform for the introduction of the overall pipeline (Fig. 1A), with the interface shown in Fig. 1B.

The major requirement for “findGSEP” is a histogram file, which can be generated using “jellyfish” (Marçais and Kingsford 2011) or other *k*-mer counting tools like “kmc” (Kokot *et al.* 2017) (usage of both *k*-mer counting tools provided at GitHub repository). The histogram file is composed of a number of tab-separated lines, where for each line, the first number gives the *k*-mer coverage (*c_i_*), while the second gives the frequency or count (*f_i_*) of *k*-mers occurring *c_i_* times, *i* = 1,2,3…. The overall pipeline of “findGSEP” is outlined in Algorithm 1 (with more details in Supplementary Note). Note, the idea underlying “findGSEP” is the same as “findGSE”; however, a minimum of *p* iterations of curve fittings around *C_het_*, 2*C_het_, …, p*C_het_ are performed, where *C_het_* is the observed *k*-mer coverage per haplotype chromosome according to the *k*-mer counting histogram. This can reveal the sizes of genomic regions shared by 1,2,…,*p* homologous chromosomes.

### 2.1 Input of “findGSEP”

To run “findGSEP” web server, a minimum set of three input parameters need to be provided at the “Home” page, i.e., the (file of) *k*-mer counting histogram for the species under consideration, the *k*-mer coverage *C* (any value satisfying 2×*C_het_* < *C* < 3 × *C_het_*), and the ploidy *p* of the species. For scaling the visualization area of the curve of the *k*-mer frequencies, the limits on *x*/*y* axis can also be set up by users. After clicking the “Submit” button, the server runs in the background to generate the genome size estimates, and visualize the observed and fitted *k*-mer frequencies.

### 2.2 Output of “findGSEP”

Outputs can be found at the “Download” page. The size estimates include the average haploid genome size (*G*), the full genome size (G*p), the sizes of genomic regions being unique to one chromosome, those common to 2∼*p* homologous chromosomes, as well as repeats. All outputs can be downloaded as a PDF file.

As introduced earlier, most existing tools for genome size estimation are challenged by polyploid genomes with *p* > 2. “GenomeScope” extends its capability in handling species with *p* up to six. While “findGSEP” was implemented without such a limitation and thus can be featured by its capability of handling species with any ploidy (Table 1).

**Table 1.** Feature of various tools for genome size estimation.

Functions	findGSEP	GenomeScope	CoVest	Kmergenie
CLI tool	Yes	Yes	Yes	Yes
Online server	Yes	Yes	/	/
Histogram upload	Yes	Yes	/	/
Histogram type-in	Yes	/	/	/
No ploidy limit	Yes	/	/	/

Algorithm 1. findGSEP

**Input**: *p*, *C*, and a file of *k*-mer count/frequency generated from whole-genome sequencing data of the species of interest

**Output**: average haploid genome size *G*

1: **For** *i* in 1: *p*

2: *Scale raw *k*-mer counts to smaller values according to *C*

3: *Fill in missing data using an approximate interpolation method

4: **Fit** *k*-mer counts shared by *i* homologous chromosomes following a normal distribution model (Sun *et al.* 2018)

5: *Scale *k*-mer counts from curve fitting to the level of raw counts

6: **If** (*i* == 1)//get *k*-mer coverage per haplotype chromosome

7:  *C_het_* = *k*-mer coverage at the peak of the curve

8: **End If**

9. **Estimate** region size as *g_i_* = sum(fitted *k*-mer counts)/(p*C_het_)

10: **End for**

11: **Estimate** repeat size *g_p_*_+1_, where *g_p_*_+1_ = sum(the remaining *k*-mer counts after the last *k*-mer fitting)/(p*C_het_)

12. **Visualize** the raw and fitted *k*-mer counts

13: **Return** genome size *G* as sum(*g_i_* | *i* = 1: (*p* + 1))

*Note 1, for “**scale**,” “**fill**” operation, see Supplementary note

## 3 Results

To demonstrate the performance of “findGSEP”, we carried out assessment by comparing it to another tool, “GenomeScope”, across a diverse set of real polyploid species and simulated data. All the histogram files and corresponding accession numbers are available in (Supplementary Table S1). We executed the CLI versions of both tools. All the parameter settings for “findGSEP” is shown in Supplementary Table S1, while “GenomeScope” was run with default parameter settings.

We first tested “findGSEP” on simulated genomes with various ploidy, using *Arabidopsis thaliana* sequences (∼135 Mb each) recently assembled by Lian *et al.* (2024). Genomes with ploidy levels ranging from 3 to 8 were simulated by combining different Arabidopsis genomes, where the total sequence length (divided by the ploidy number) was taken as the true genome size, each with three replicates. Short reads were then simulated from these polyploid genomes using the pirs (Hu *et al.* 2012). “Jellyfish” was used to generate *k*-mer frequency histogram files from the simulated short reads. These histograms were then taken by “findGSEP” to predict the genome size. The estimated sizes were highly consistent to the true sizes, as shown in Supplementary Fig. S15 and Supplementary Table S2, demonstrating that our method accurately predicts genome sizes for polyploid genomes, particularly in comparison with “GenomeScope,” which cannot achieve accurate estimations for species with ploidy over six (Supplementary Fig. S16).

We then analyse on the real data for various species. Results showed that “findGSEP” tends to produce larger size estimates than “GenomeScope” (Fig. 1C), where the differences can be significant, e.g. for crayfish, up to 1000 Mb. The size estimates given “GenomeScope” were sometimes even lower than the assembly sizes (e.g. for potato), which indicated that it might have underestimated the genome size. In contrast, the estimates of “findGSEP” were closer to the assembly sizes than “GenomeScope,” for seven out of nine species (Fig. 1C, with name of species in red). On the other hand, the assembly sizes of *enterolobii*, *incognita*, and *javanica* were larger than the estimates by both tools, probably indicating that there were a high percentage of haplotype-resolved contigs. Particularly, for octoploid strawberry and Chinese sturgeon (Wang *et al.* 2024), only “findGSEP” was able to deliver an accurate estimation of 207 and 905 Mb, while “GenomeScope” gave 667 and 586 Mb (Fig. 1D and Supplementary Fig. S17).

Also, we evaluated time-efficiency of both methods with the genome sequencing data of diploid, polyploid species (*p* from 2 to 6). “findGSEP” showed higher efficiency with respective to ploidy values than “GenomeScope” (Fig. 1E and Supplementary Table S3).

## 4 Discussion

“findGSEP” was developed to provide an easy-to-use interactive tool for estimating genome size using *k*-mer frequencies. The tool works for species with any ploidy, and as far as we know, it is the only tool that can perform the task for species with ploidy higher than six. “findGSEP” offers flexibility in usage, either through a standalone version of the *R* package or an online web server, and produces publication-quality figures. Currently, it still requires a user to input a ploidy estimate. To leave out this requirement, one related project is ongoing, aiming to further enhance the tool with automatic ploidy number estimation.

## Supplementary Material

btae647_Supplementary_Data
