# Supplementary material for: findGSEP: estimating genome size of polyploid species using k-mer frequencies
Source: Bioinformatics. 2024 Oct 30;40(11):btae647. doi: 10.1093/bioinformatics/btae647 (PMC11552620; doi:10.1093/bioinformatics/btae647)
Supplement: btae647_Supplementary_Data [file btae647_supplementary_data.pdf]

Supplementary information

findGSEP: Estimating genome size of polyploid species using *k*-mer frequencies

Laiyi Fu[1,2,3], Yanxin Xie[1], Shunkang Ling[4], Ying Wang[1], Binzhong Wang[5], Hejun Du[5], Qinke Peng[1], and Hequan Sun[1,6,*]

[1]School of Automation Science and Engineering, Faculty of Electronic and Information Engineering, Xi'an Jiaotong University, 710049 Xi'an, China

[2]Research Institute of Xi'an Jiaotong University, Zhejiang, 311200 Hangzhou, China

[3]Sichuan Digital Economy Industry Development Research Institute, 610036 Chengdu, China

[4]College of Mechanical and Electrical Engineering, Shihezi University, 832000 Shihezi, China

[5]Hubei Key Laboratory of Three Gorges Project for Conservation of Fishes, Yichang, Hubei, 443100, China

[6]Department of Chromosome Biology, Max Planck Institute for Plant Breeding Research, Carl-von-Linné-Weg 10, 50829 Cologne, Germany

[*]Correspondence: hequan.sun@xjtu.edu.cn.

Supplementary Notes

Algorithms underlying *findGSEP*

Scale and Rescale

We first calculate scaling factor with raw *k*-mer counts data *X*: (c_i, f_i), *i*=1,2,3,…, where c_i gives the *k*-mer coverage (c_i), while f_i gives the frequency of *k*-mers occurring at coverage c_i, according to the *k*-mer coverage *C* (any value satisfying $2*C_{het} < C < 3*C_{het}$ where C_{het} is the observed *k*-mer coverage per haplotype chromosome from the histogram). If *C* is greater than or equal to 80, the scaling factor is calculated as *scaled_factor* = *C* / 60. Then, the *k*-mer frequencies are scaled as follows: $c'_i = c_i$ / scaled_factor and $f_i' = f_i$ * scaled_factor.

Rescale after curve fitting

After fitting each ploidy curve, rescaling procedure is performed to get the final fitted counts at raw data scale. $c_i = c'_i$ * scaled_factor and $f_i = f_i'$ / scaled_factor.

Data interpolation

We employed linear interpolation to fill the missing data after scale and rescale procedure. This estimation is achieved through interpolation, where the value of c_i falls between the two nearest input values in c_i:c_{i+1}. Given input data *X*: (c_i, f_i), where $C = [c_i, c_2, ..., c_n]$ and the corresponding output data $F = [f_1, f_2, ..., f_n]$, the interpolation method estimates the target output value f_{out} as follows:

1. Find the two nearest input values c_i and c_{i+1} in the input data X, where $c_i <= c_{out} <= c_{i+1}$.
2. Calculate the relative position of c_{out} between c_i and c_{i+1}, denoted as t:
   $$t = (c_{out} - c_i) / (c_{i+1} - c_i)$$
3. Use the weight corresponding to t to estimate the target output value f_{out}:
   $$f_{out} = (1 - t) * f_i + t * f_{i+1}$$

Contamination in DNA sample

Contamination in DNA sampling is unavoidable in some cases. If the amount of DNA from contamination is comparable to or more than the target genome, it is challenging to distinguish the corresponding *k*-mers from those of the target genome in the *k*-mer frequency distribution. If the amount of contamination is minor, this will result in an additional small peak in the *k*-mer frequency distribution, except for peaks formed by *k*-mers from the target genome. The contamination peak will be located next to the peak representing for the sequencing errors (besides y-axis). This might mislead *findGSEP* in determining the heterozygous peak. To avoid this, if there is an indication that DNA sampling could have been contaminated, it is better filtering the sequencing reads. For example, extract a tiny portion of sequencing reads randomly from the whole read set, blast them against the NCBI nucleotide database, and determine the contamination source (i.e., species). Then download the full genome(s) (referred to as *G*) of the species considered as contamination, align all sequencing reads to *G*, and keep only reads that could not be aligned. Alternatively, if it is clear which peak in the k-mer frequency distribution represents *k*-mers from *G*, the surrounding *k*-mer frequency can be set as 0 manually. This will not affect the accuracy in genome size estimation, as *findGSEP* will calculate the *k*-mer frequency for such cases automatically through the *k*-mer fitting process.

Workflow of *findGSEP*

To use *findGSEP*, two main steps are needed, including generating histogram file using either *KMC* or *Jellyfish* tools, and running *findGSEP*. To run the pipeline below, the versions of tools include: *Jellyfish* 2.3.0, *KMC* 3.2.1 (2022-01-04) and *R* 4.2.0, and *findGSEP*_1.2.0. *R* Package Versions include *dplyr_1.1.4, png_0.1-8, scales_1.3.0, fGarch_4033.92, pracma_2.4.4, ggplot2_3.5.0*, and *RColorBrewer_1.1-3*.

Step 1. Generate histogram file using either *KMC* or *Jellyfish* tools

1.1 Instructions for running *Jellyfish*
1.1.1 Download and install jellyfish from *http://www.genome.umd.edu/jellyfish.html#Release*
1.1.2 Count k-mers using *Jellyfish*

```
jellyfish count -C -m 21 -t 1 -s 5G *.fastq -o reads.mer
```

1.1.3. Export the *k*-mer count histogram

```
jellyfish histo -h 3000000 -t 10 -o reads.histo reads.mer
```

1.1.4. Upload reads.histo to *findGSEP* (details given in the following).

1.2 Instructions for running *KMC*
1.2.1 Download and install *KMC* from *https://github.com/refresh-bio/KMC*
1.2.2 Count kmers using *KMC*

```
kmc -k21 -fa -t50 -m12 -ci1 @input_file_name.lst reads_kmc tmp
```

Note: input_file_name.lst is the file name list with locations of files of sequencing reads. -fa means input file is fasta format data. Adjust the memory (-m) and threads (-t) parameters according to your server. This example uses 50 thread and 12GB of RAM as default. For other parameters, please refer to the manual of the tool itself.

1.2.3 Export the *k*-mer count histogram

```
kmc_tools transform reads_kmc histogram reads_kmc.histo
```

1.2.4 Upload reads_kmc.histo to *findGSEP*

Step 2. Run *findGSEP*

2.1 Instructions for installing *findGSEP*

Get the released version from CRAN:

```
install.packages("findGSEP")
```

Or the development version from github:

2.1.1 Install devtools

```
install.packages("devtools")
```

2.1.2. Install directly from GitHub:

```
devtools::install_github("sperfu/findGSEP")
```

Note: This package was developed using *R* version 4.2.0. To ensure the stability of the package, it is highly recommended that users install *R* version 4.2.0.

2.2 Instructions for retrieving Data

All histogram files used have been provided at *http://146.56.237.198:3838/findGSEP/* or online repositories such as Zenodo: *10.5281/zenodo.13147431 and Google drive: https://drive.google.com/drive/folders/1fBuPRxi_J-oMpj6G2KokEUeB_8S8ahH6?usp=share_link, with histogram files for species of tetraploid to octoploid.*

2.3 Instructions for running *findGSEP*

Taking a simulated pentaploid genome as an example, suppose the histogram file is your_file.histo, in the *R* environment, type in the following commands from the command line:

```
# Set options (optional):
options(warn = -1)
# Define input parameters:
path <- "histo_files"
samples <- "your_file.histo"
sizek <- 21
exp_hom <- 200
ploidy <- 4
output_dir <- "outfiles"
xlimit <- -1
ylimit <- -1
range_left <- exp_hom * 0.2
range_right <- exp_hom * 0.2
# Call the findGSEP function with specified parameters:
findGSEP(path, samples, sizek, exp_hom, ploidy, range_left, range_right, xlimit,
ylimit, output_dir)
```

When finished, *your_file.histo_hap_genome_size_est.pdf* can be found in the *output_dir* folder.

Supplementary Figures

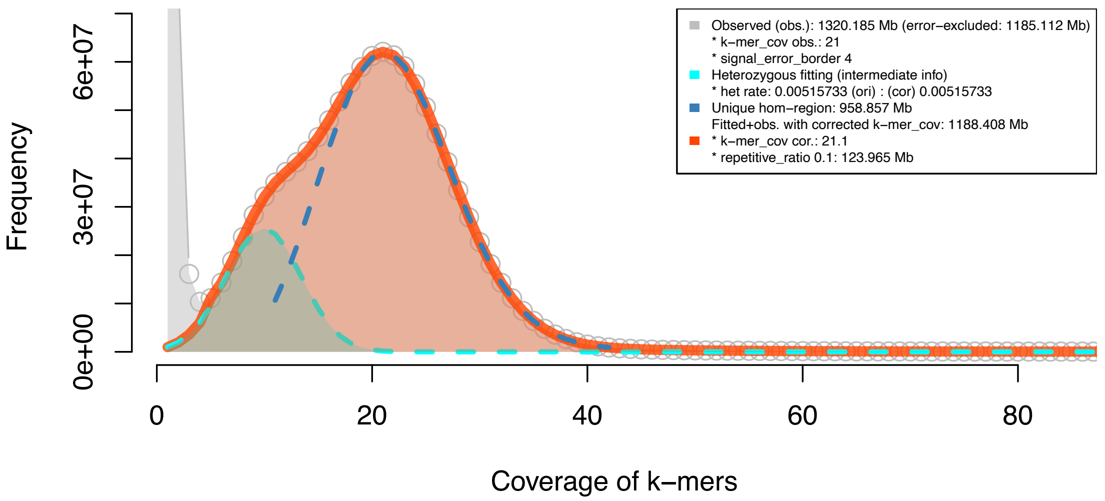

Supplementary Fig. S1. Bird (Budgerigar, diploid) (Aleksey V. Zimin *et al.*, 2017; Ranallo-Benavidez *et al.*, 2020)

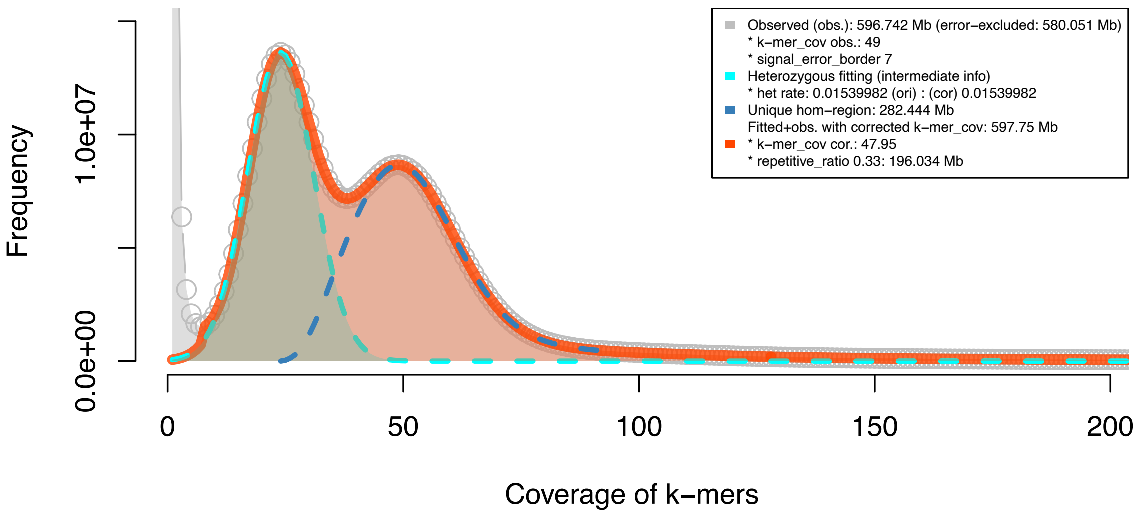

Supplementary Fig. S2. Oyster (diploid) (Ranallo-Benavidez *et al.*, 2020)

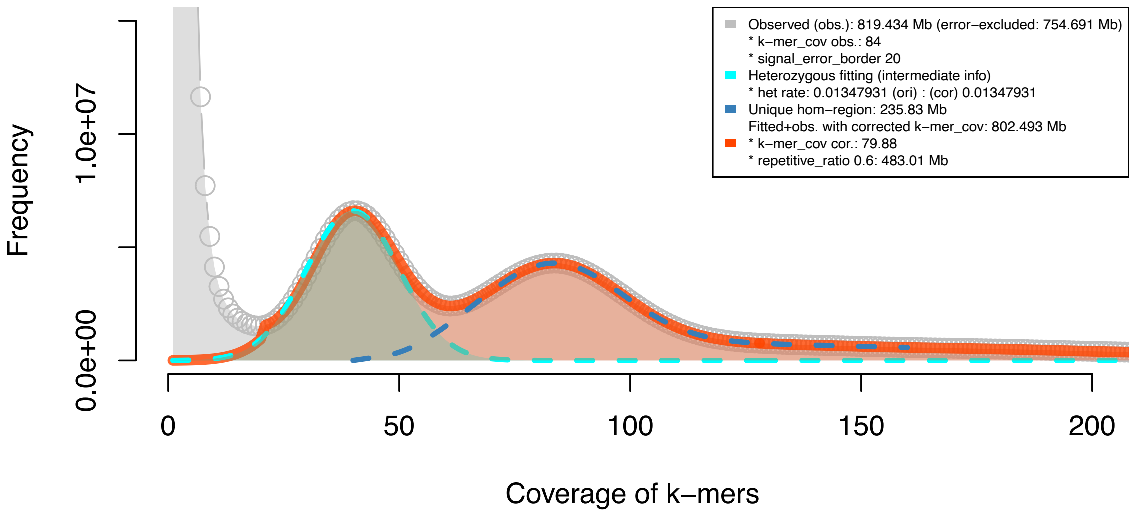

Supplementary Fig. S3. Pear (diploid) (Ranallo-Benavidez *et al.*, 2020)

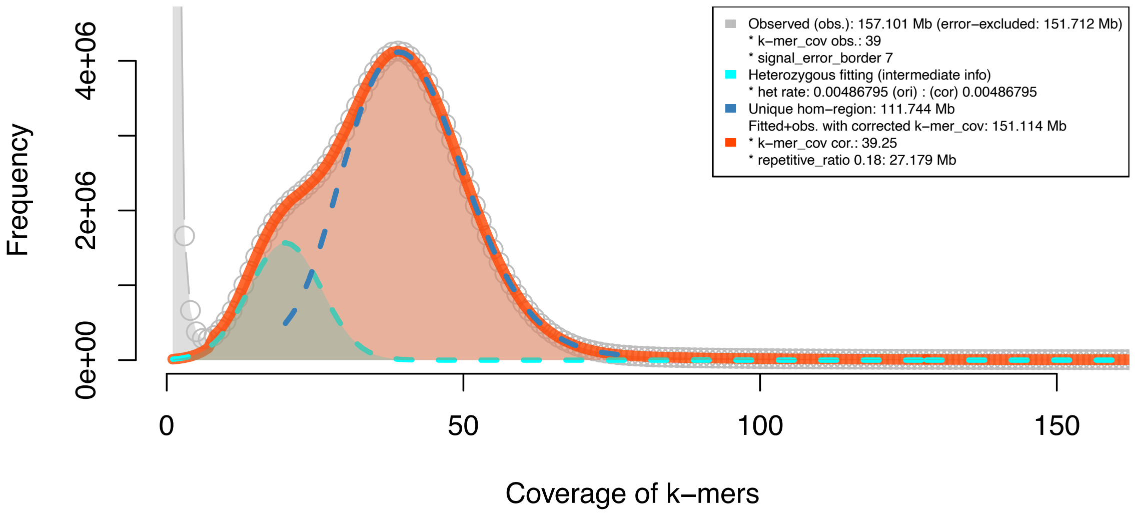

Supplementary Fig. S4. Drosophila (diploid) (Ranallo-Benavidez *et al.*, 2020)

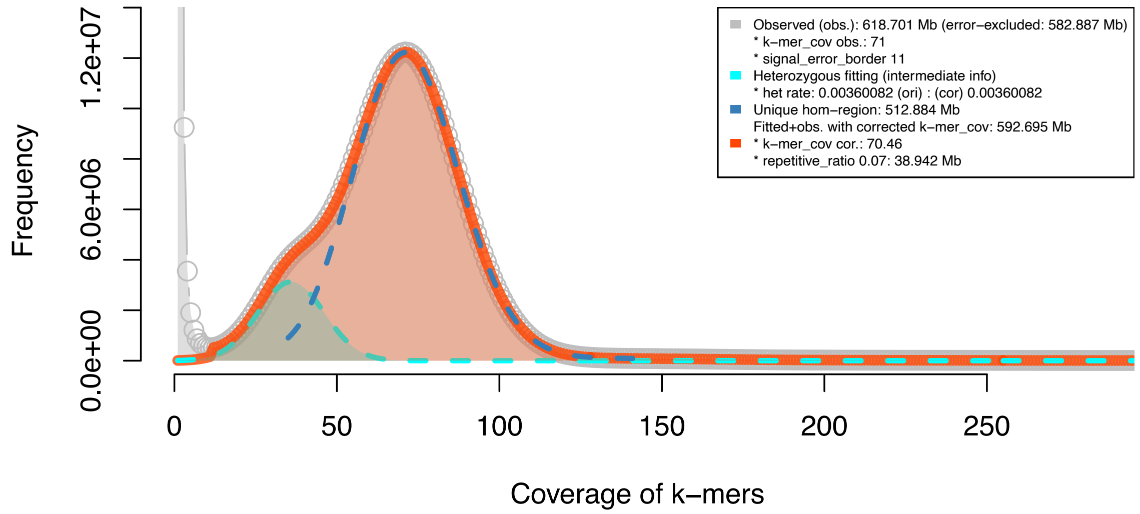

Supplementary Fig. S5. Seabass (diploid) (Ranallo-Benavidez *et al.*, 2020)

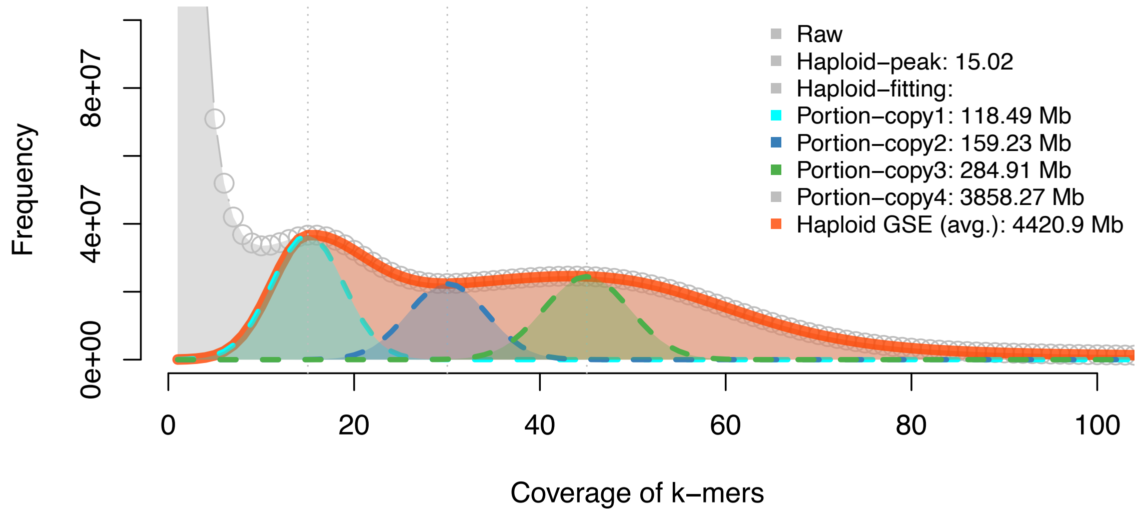

Supplementary Fig. S6. Crayfish virginalis (triploid) (Gutekunst *et al.*, 2018)

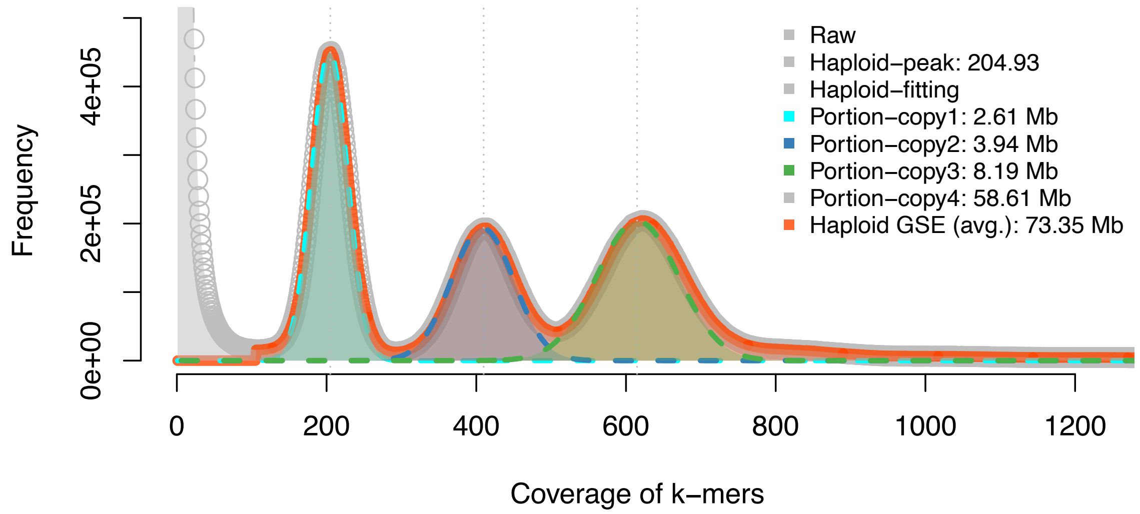

Supplementary Fig. S7. Meloidogyne floridensis (triploid) (Szitenberg *et al.*, 2017)

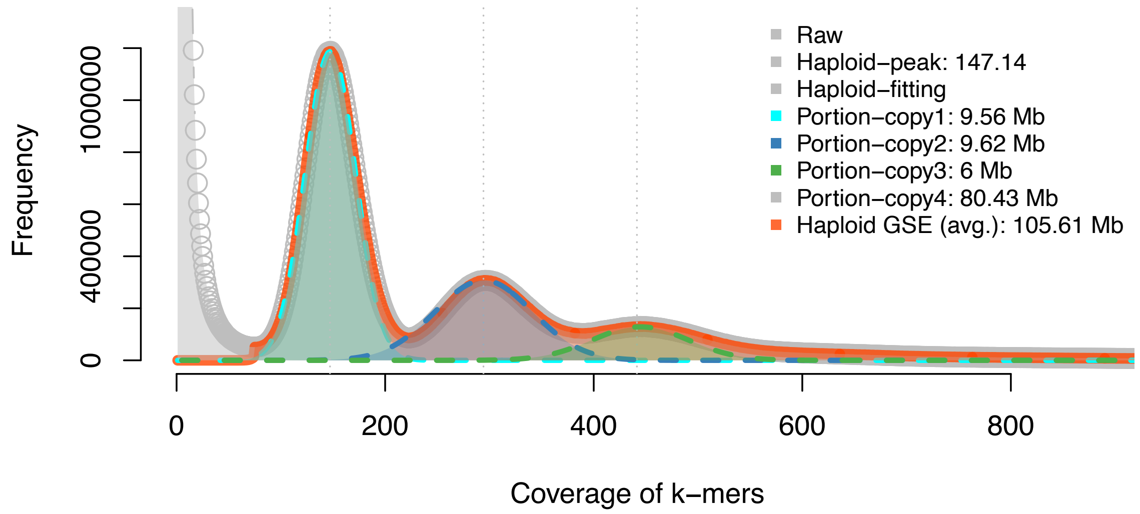

Supplementary Fig. S8. Meloidogyne enterolobii (triploid) (Szitenberg *et al.*, 2017)

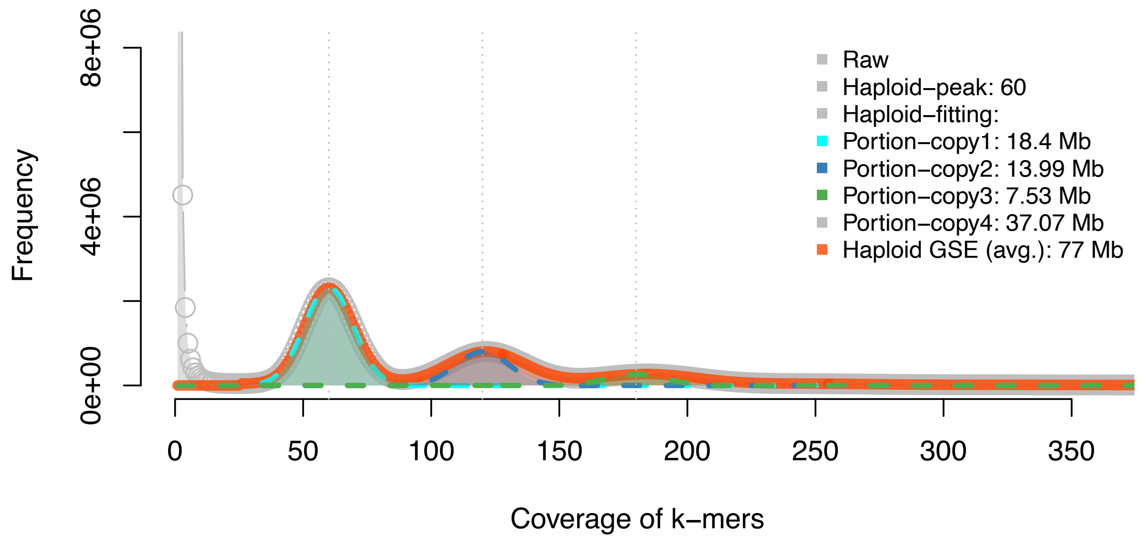

Supplementary Fig. S9. Meloidogyne incognita (triploid) (Szitenberg *et al.*, 2017)

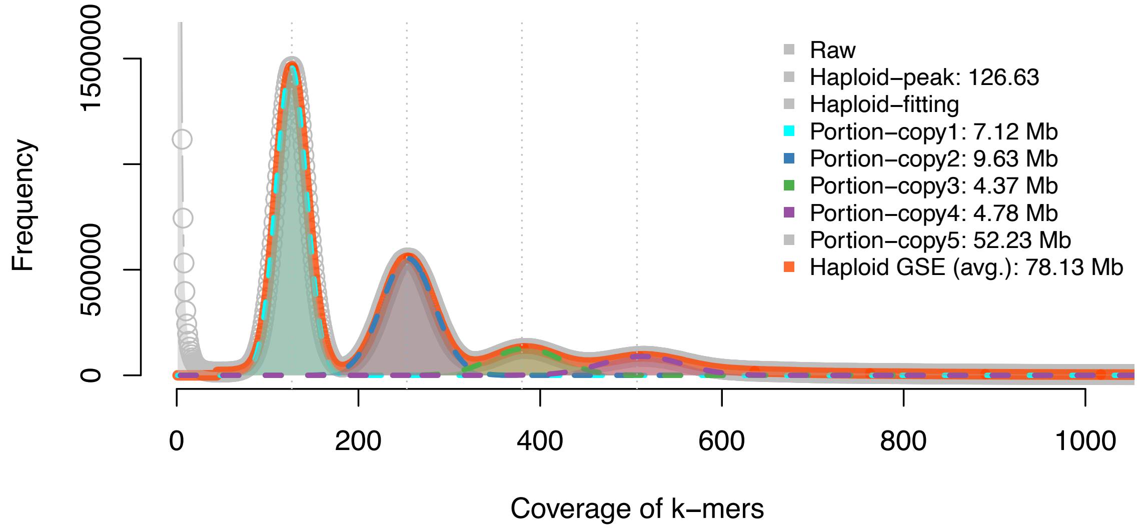

Supplementary Fig. S10. Meloidogyne javanica (tetraploid) (Szitenberg *et al.*, 2017)

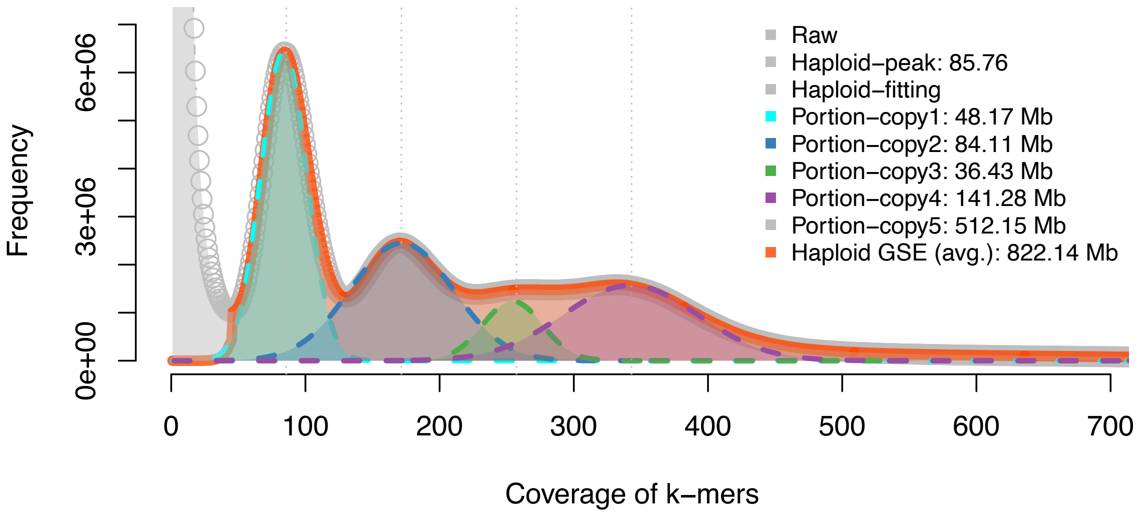

Supplementary Fig. S11. Potato (tetraploid) (Sun *et al.*, 2022)

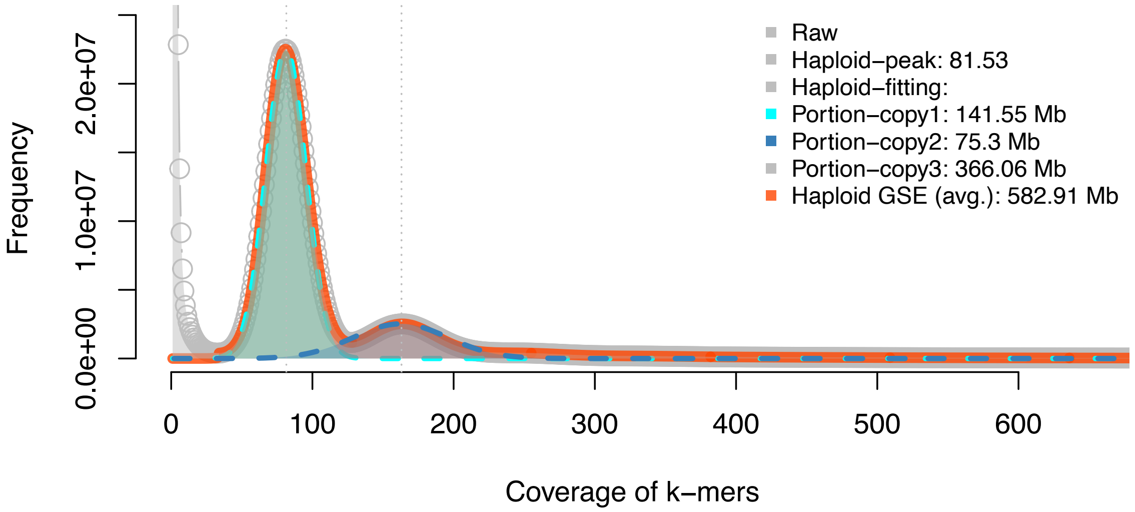

Supplementary Fig. S12. Cotton (Gossypium barbadense) (tetraploid) (Wang *et al.*, 2019)

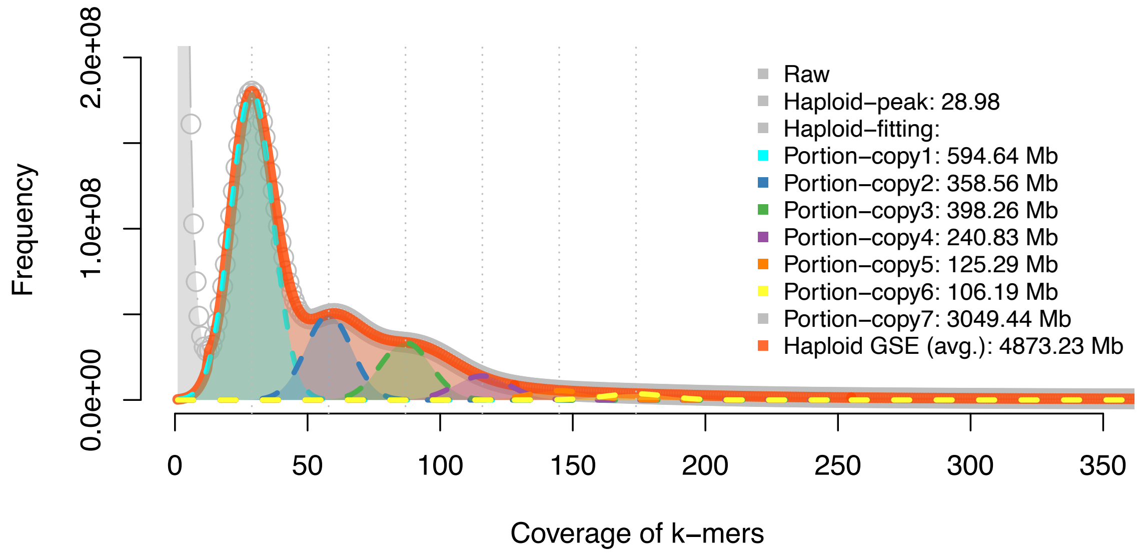

Supplementary Fig. S13. Coastal redwood (Sequoia sempervirens) (hexaploid) (Ranallo-Benavidez *et al.*, 2020)

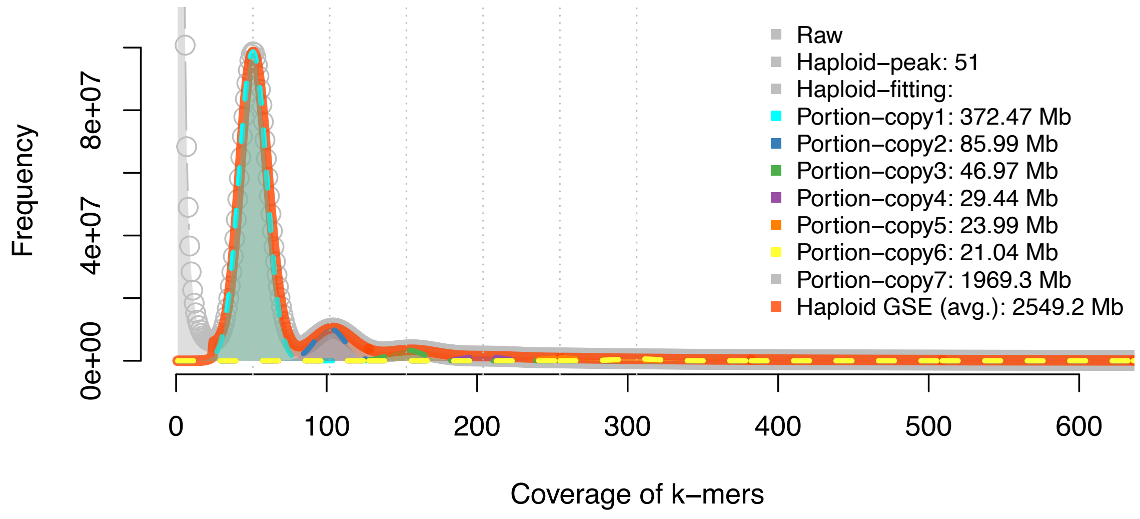

Supplementary Fig. S14. wheat (hexaploid) (Aleksey V Zimin *et al.*, 2017)

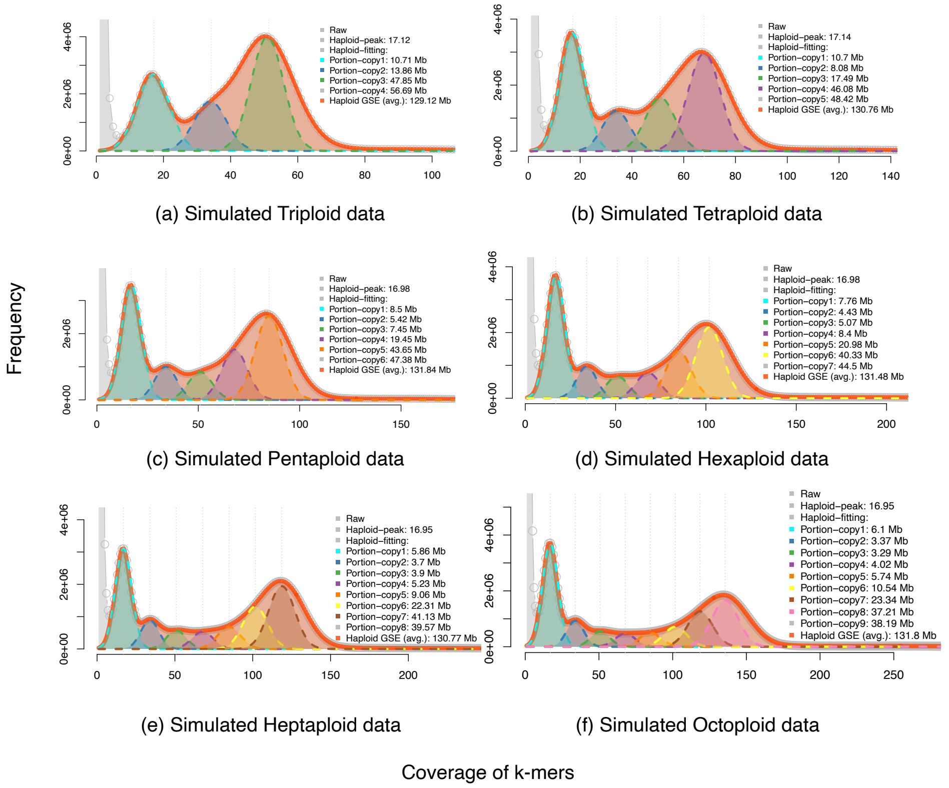

Supplementary Fig. S15. Visualization examples of *findGSEP* predictions on simulated genomes of Triploid to Octoploid(Lian *et al.*, 2024).

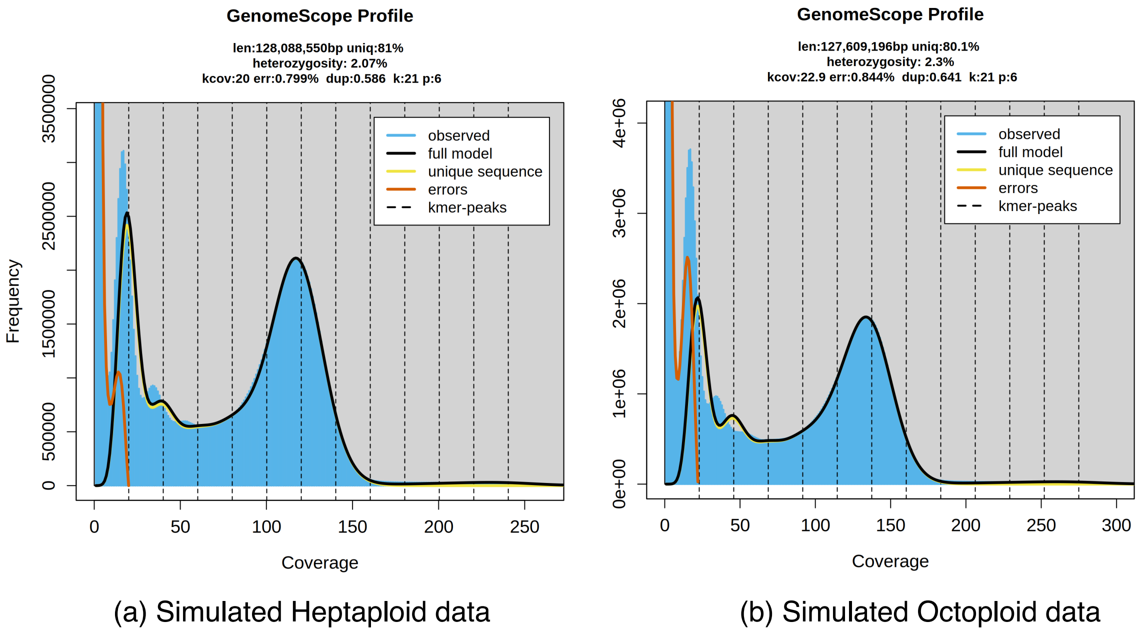

Supplementary Fig. S16. Visualization examples of *GenomeScope* predictions on simulated hexaploid and octoploid genomes.

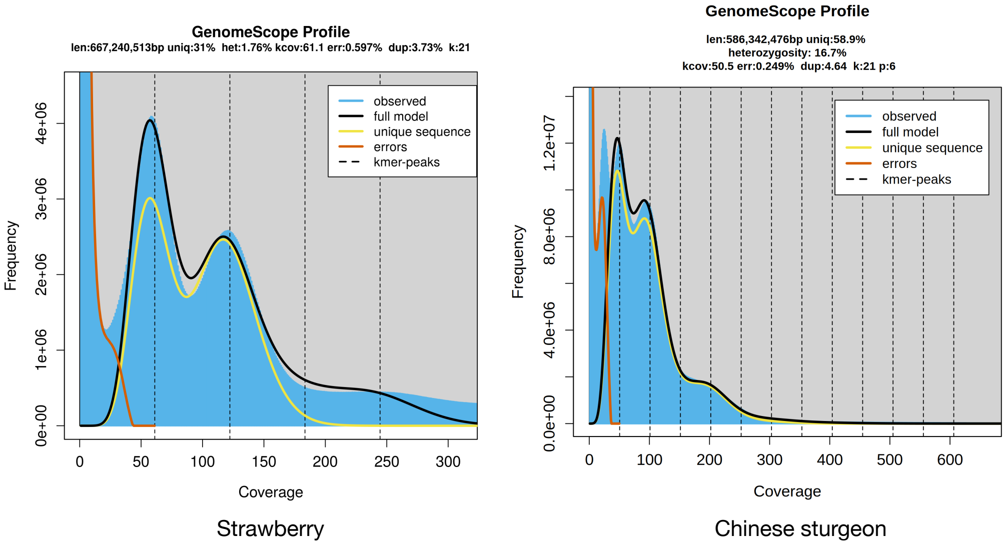

Supplementary Fig. S17. *GenomeScope* predictions on Strawberry and Chinese sturgeon (Wang *et al.*, 2023)

Supplementary Tables

Table S1 Dataset and key parameter settings for running *findGSEP*

Species	Expected Hom	Ploidy number	Size *k*	Accession number	Database
Chinese sturgeon	100	8	21	CRA009603	GSA
Strawberry	100	8	21	PRJDB3320	NCBI
Wheat	150	6	21	SRX2994097	NCBI
Redwood	80	6	21	PRJNA542879	NCBI
Cotton	150	4	21	SRX959277	NCBI
Javanica	200	4	21	SRX2163385	NCBI
Potato	180	4	21	SRX2645997	NCBI
Floridensis	220	4	21	SRX2163402	NCBI
Crayfish	35	3	21	SRX2417036	NCBI
Enterolobii	130	3	21	SRX2163399	NCBI
Incognita	200	3	21	SRX2163387	NCBI
Seabass	80	2	21	SRP069219	NCBI
Budgerigar(Bird)	40	2	21	ERR244146	NCBI
Drosophila	50	2	21	Histogram file from literature	(Keightley et al., 2014; T. Ranallo-Benavidez et al., 2020)
Pear	100	2	21	SRP016889	NCBI
Oyster	50	2	21	SRP045757	NCBI

Table S2 Prediction by *findGSEP* using simulated datasets

Ploidy number	Combined accessions of *Arabidopsis Thaliana* (Lian et al., 2024)	True size	Predicted size
3	Bay0, Ge-0, Ri-0	130Mb	130Mb
3	Ri-0, Are-10, JEA	131Mb	131Mb
3	Ri-0, Shigu-2, JEA	129Mb	129Mb
4	JEA, Bay-0, Are-10, Oy-0	132Mb	132Mb
4	Ri-0, JEA, Blh-1, Bay-0	129Mb	129Mb
4	JEA, Yo-0, Bay-0, Are-10	132Mb	132Mb
5	Ge-0, Ri-0, Are-10, Oy-0, Bay-0	132Mb	132Mb
5	Oy-0, Ge-0, Bay-0, Ri-0, Shigu-2	131Mb	131Mb
5	Bay-0, JEA, Are-10, Ge-0, Shigu-2	132Mb	132Mb
6	Bay-0, JEA, Are-10, Ri-0, Shigu-2, Oy-0	131Mb	131Mb
6	Ge-0, Ri-0, Shigu-2, Bay-0, JEA, Oy-0	131Mb	130Mb
6	Ge-0, Ri-0, Shigu-2, Bay-0, JEA , Are-10	131Mb	131Mb
7	Shigu-2, Are-10, Bay-0, Oy-0, Ri-0, JEA, Yo-0	131Mb	131Mb
7	Bay-0, JEA, Are-10, Ri-0, Shigu-2, Oy-0, Ge-0	131Mb	132Mb
7	Ge-0, Ri-0, Shigu-2, Bay-0, JEA, Blh-1, Oy-0	131Mb	131Mb
8	Yo-0, JEA, Blh-1, Bay-0, Ri-0, Oy-0, Bur-0, Are-10	131Mb	132Mb
8	Ri-0, Oy-0, Ge-0, Bur-0, Are-10, Bay-0, Blh-1, JEA	131Mb	132Mb
8	Bur-0, Are-10, Blh-1, JEA, Ri-0, Bay-0, Yo-0, Ge-0	131Mb	132Mb

Table S3 Computational resources

Species	Size of read set (Gb)	RunTime (Hour)			Memory usage (Gb)			Disk usage (Gb)		Ploidy number
		KMC	*Jellyfish*	*findGSEP*	*KMC*	*Jellyfish*	*findGSEP*	Jellyfish	KMC	
Chinese sturgeon	314.4	2.47	14.90	0.091	12	49	5.00	59	47.5	8
Strawberry	144.7	1.54	11.10	0.075	12	49	2.90	87	50	8
Wheat	942.5	13.0	94.14	0.100	12	87	5.50	257	204.1	6
Cotton	247.5	2.87	16.62	0.011	12	46	1.49	66	52.1	4
Javanica	48.1	0.55	3.37	0.005	12	49	0.64	7.4	9.2	4
Potato	2.1	0.03	0.14	0.044	12	46	3.81	1.1	2.54	4
Crayfish	245.2	3.06	17.20	0.024	12	49	5.88	92	62.5	3
Incognita	17.2	0.19	1.21	0.009	12	44	1.78	5.4	20.8	3
Simulation (Triploid)	9.75	0.13	0.66	0.003	12	30	0.70	12	15.9	3
Simulation (Tetraploid)	13.0	0.16	1.00	0.005	12	32	0.84	15	21.0	4
Simulation (Pentaploid)	16.5	0.21	1.20	0.006	12	44	0.92	18	25.6	5
Simulation (Hexaploid)	19.75	0.23	1.51	0.007	12	46	0.99	21	31.4	6
Simulation (Heptaploid)	23.0	0.30	1.59	0.008	12	46	1.10	23	35.8	7
Simulation (Octoploid)	26.25	0.32	1.83	0.012	12	46	1.20	26	40.2	8

Supplementary References

Gutekunst,J. *et al.* (2018) Clonal genome evolution and rapid invasive spread of the marbled crayfish. *Nat. Ecol. Evol.*, **2**, 567–573.

Keightley,P.D. *et al.* (2014) Estimation of the spontaneous mutation rate per nucleotide site in a Drosophila melanogaster full-sib family. *Genetics*, **196**, 313–320.

Lian,Q. *et al.* (2024) A pan-genome of 69 Arabidopsis thaliana accessions reveals a conserved genome structure throughout the global species range. *Nat. Genet.*, 1–10.

Ranallo-Benavidez,T.R. *et al.* (2020) GenomeScope 2.0 and Smudgeplot for reference-free profiling of polyploid genomes. *Nat. Commun.*, **11**, 1432.

Sun,H. *et al.* (2022) Chromosome-scale and haplotype-resolved genome assembly of a tetraploid potato cultivar. *Nat. Genet.*, **54**, 342–348.

Szitenberg,A. *et al.* (2017) Comparative Genomics of Apomictic Root-Knot Nematodes: Hybridization, Ploidy, and Dynamic Genome Change. *Genome Biol. Evol.*, **9**, 2844–2861.

Wang,B. *et al.* (2023) Whole-genome Sequencing Reveals Autooctoploidy in Chinese Sturgeon and Its Evolutionary Trajectories. *Genomics Proteomics Bioinformatics*, qzad002.

Wang,M. *et al.* (2019) Reference genome sequences of two cultivated allotetraploid cottons, Gossypium hirsutum and Gossypium barbadense. *Nat. Genet.*, **51**, 224–229.

Zimin,Aleksey V. *et al.* (2017) The first near-complete assembly of the hexaploid bread wheat genome, Triticum aestivum. *GigaScience*, **6**, 1–7.
